# The role of demographic and academic features in a student performance prediction

**DOI:** 10.1038/s41598-022-15880-6

**Published:** 2022-07-22

**Authors:** Muhammad Bilal, Muhammad Omar, Waheed Anwar, Rahat H. Bokhari, Gyu Sang Choi

**Affiliations:** 1 Department of Computer Science & IT, The Islamia University of Bahawalpur, Bahawalpur, Pakistan; 2 Department of Data Science, Faculty of Computing, The Islamia University of Bahawalpur, Bahawalpur, Pakistan; 3 Department of Computer Science, Faculty of Computing, The Islamia University of Bahawalpur, Bahawalpur, Pakistan; 4 Department of Computer Science, University of South Asia, Lahore, Pakistan; 5 Department of Information and Communication Engineering, Yeungnam University, 280 Daehak-Ro, Gyeongsan-si, 38541 South Korea

**Keywords:** Computer science, Information technology

## Abstract

Educational Data Mining is widely used for predicting student's performance. It’s a challenging task because a plethora of features related to demographics, personality traits, socio-economic, and environmental may affect students' performance. Such varying features may depend on the level of study, program offered, nature of subject, and geographical location. This study attempted to predict the final semester’s results of students studying Doctor of Veterinary Medicine (DVM) based on their pre-admission academic achievements, demographics, and first semester performance. The imbalanced data led to non-generic prediction models, so it was addressed through synthetic minority oversampling technique. Among five prediction models, the Support Vector Machine led the best with 92% accuracy. The decision tree model identified key features affecting students’ performance. The analysis led to the conclusion that marks obtained in Biology, Islamiat, and Urdu at Matric and English at Intermediate level affected the students’ performance in their final semester. The findings provide useful information to predict students’ performance and guidelines for academic institutes’ management regarding improving students’ achievement. It is speculated that adoption of digital transformation may help reduce difficulty faced in data collection and analysis.

## Introduction

A higher education institute aims to provide a quality education to the students for achieving outstanding performance on their part. Students’ academic performance is the most important quality measure that depends on several factors such as demographics, personality traits, socio-economic, and other environmental factors. The knowledge about these factors and their effect on students’ performance can assist in managing their impact. Educational institutes are generating a large volume of data related to students studying in degree programs. The data generated at institute levels may be further transformed and analysed leading to meaningful information that may assist faculty, administration, and policymakers to make decisions regarding institutional matters and particularly the students and their well-being. Predicting students’ academic performance has long been a significant research area in educational institutes and become a challenging task due to large number performance affecting factors^1^.

Data mining methods are used to get meaningful information and hidden patterns from data and the application of data mining methods to educational data is called Educational Data Mining (EDM)^2–4^. Data mining is one of the most famous technique to evaluate academic performance^5^. Artificial intelligence (AI), data mining, and data science are overlapping fields where machine learning algorithms are used to learn from the data without being explicitly programmed. Students’ academic performance prediction with the help of supervised machine learning models is an important application in EDM. According to literature (see next section), students’ academic performance prediction has been performed at different levels: subjects^6–9^, semester^10–13^, and degree grade level^14–16^. The current work investigates final semester (10th semester) performance prediction (high and low performance) of a student at an early stage, more specifically after first semester of DVM degree program.

The study addresses the following research questions:

RQ1: Can we predict the final semester performance of a Doctor of Veterinary Medicine (DVM) student with high accuracy based on pre-admission features and first-semester performance?
RQ2: What are the features that affect the final semester performance of the DVM student?

The results show that we can predict performance with high accuracy and subsequently find key performance affecting features. This research may help the faculty to promote better students and to provide additional teaching support for low performers by taking into account the most important features that affect students’ academic performance. Administration can consider these effective features for student counselling to adjust admission criteria and to enhance the admission decision-making process based on these effective features.

## Literature review

Students’ performance prediction has been performed at different levels: single subject level in terms of marks, semester level in terms of SGPA, and degree level in terms of overall grade, average percentage marks or CGPA.

At the subject level, the authors have predicted the marks of the Introduction to informatics module of distance learning at Hellenic Open University, Greece using demographic features/variables (age, sex, and occupation, etc.), assignment marks, and face to face meetings^6^. The Study^7^ used cognitive features (CGPA, Pre-requisites courses’ marks, and midterm marks) to predict the undergraduate’s performance of engineering dynamic course at Utah State University, Logan, USA. In other studies^8,9^ the authors predicted performance (fail/pass) in core courses using cognitive features (progressive, past performance, CGPA), and using observations based on in and on-campus activities.

At the semester level (also focus of this study), the authors in^10^ predicted whether a student will pass or fail at the end of the semester using student academic information, student activity, and student video interactions. Another study^11^ performed experiments to predict semester GPA (SGPA) using quizzes, discussion, assignments, attendance, and lab work . Pre-university characteristics and previous academic performance were used^12^ to predict SGPA^13^ predicted overall performance using grades of the previous four semesters.

The study^17^ conducted experiments on a sample of 250 students with 25 attributes to predict 3rd-semester performance (excellent, above average, average, or below average) using Decision Tree with 94.40% accuracy. Another study^18^ investigated the sample of 300 students to predict final semester performance and to find the features that affect semester performance using various supervised machine learning algorithms. The results showed that Random Forest outperformed other classifiers in terms of accuracy. The study conducted by^12^ investigated the relationship between social factors and academic performance to predict third-semester students’ performance. Parents’ education, and 2nd-semester performance, were good predictor. In study^10^, performance of 772 registered students in E-commerce and E-commerce technologies modules, was predicted at the end of the semester using video learning analytics where Random Forest achieved 88.30% accuracy. The state-of-the-art algorithms in^19^ were compared to predict final exam performance using demographic, student engagement, and past performance. Artificial Neural Networks (ANN) algorithm achieved high precision using student engagement and past performance whereas demographic features were reported as not significant. Unsupervised clustering algorithm K-mean and Naïve Bayes classification algorithms were used to predict student academic performance at the end of the semester using attendance, discussion, and assignment variables^11^. A naïve Bayes algorithm was used to predict students’ performance in terms of grades in the semester exam with the aid of seven features. The finding of the study was that the teachers can take essential steps to improve the performance of students whose performance was not satisfactory^20^. Another study^21^ performed experiments on a sample of 491 students’ of Maktab Rendah Sains MARA (MRSM) Kuala Berang using Naïve Bayes to predict performance of students at an early stage (2nd semester) with 74% accuracy. In^22^, Artificial Neural Network (ANN) algorithm was trained to predict the 8th-semester performance of electrical engineering students of Universiti Teknologi MARA (UiTM)., Malaysia. Correlation coefficient and Mean Square Error were used as the performance measures. The results showed that the subjects of the 1st and 3rd semesters had strong relationship with final CGPA. Based on existing e-learning methods, behavior classification based E-learning Performance (BCEP) model and process behaviour classification (PBC) model were proposed by^23^. The experiments were conducted on Open University Learning Analytics Dataset (OULAD) to predict e-learning performance and the results showed that the proposed models were performed better than the traditional methods. The objective of study^24^ was to predict poor-performing students at the end of the semester and identifying the factors that can lead students to poor performance.

The studies^14–16^ conducted experiments to predict students’ performance at degree level: electronics engineering, computer science, and civil engineering programs respectively.

The literature review shows that performance affecting features of different courses, semester and degree program can be different and there is a need to investigate performance affecting features at local levels.

### Students’ performance prediction approach

**Figure 1:**
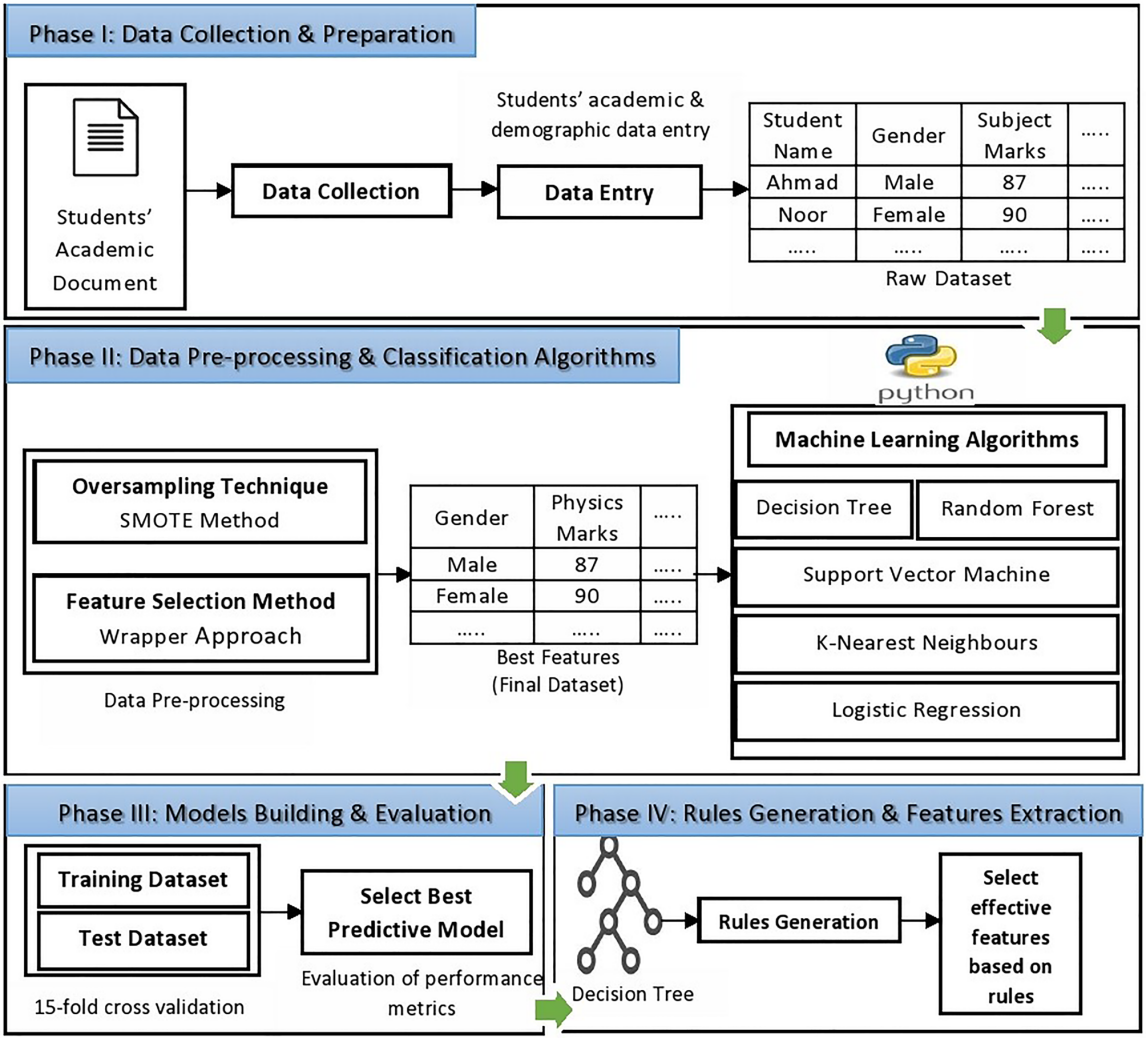
Proposed approach for student performance prediction and feature extraction.

The proposed approach comprises of four main phases (see Fig. 1). The input of our proposed approach contains students’ demographic features and pre-admission academic subjects’ marks. The dataset was imbalanced that can lead to non-generalized machine learning model (aka over fitted model). We applied Synthetic Minority Oversampling Technique (SMOTE), to overcome this problem. Then, we developed various predictive models by considering k-fold cross validation and optimally selected features. Finally, rules extracted from a decision tree model were used to explore features that can affect students’ performance. The detail of each phase is given in the following subsections.

### Data collection and storage

Due to non-digitization of the institute, most of the data was scattered in different departments and unstructured in the form of hard copies of student admission forms, and photocopies of academic certificates (matric, intermediate), national id cards etc. The percentage of the first semester SGPA and target variable (final semester SGPA) data were available at examination section in the form of Excel sheets. A formal approval to collect the data and to perform the experiments was availed from examination department, admission section, chairman of the department, and dean of the faculty. The data of 166 students was collected from three sessions: 2010–15, 2012–17, and 2013–18, of a five year Doctor of Veterinary Medicine (DVM) program of Faculty of Veterinary & Animal Sciences, The Islamia University of Bahawalpur, Punjab, Pakistan. We were not able to find the data for the admission cohort 2011–16. Though parents’ education is an important feature^12^, but most of the students didn’t provided this information so the feature was not considered in the experiments. The dataset consists of students’ demographic features, High School Certificate (HSC) subjects marks, Higher Secondary School Certificate (HSSC) subjects marks, and first semester SGPA of DVM program. The dataset was stored in an Excel file, and description of each features is documented in Table 1.

**Table 1:** Dataset variables and their metadata.

No	Features’ type	Features with description	Category	Values
1	Demographic	Gender	Categorical	Male/Female
2		Father’s Profession	Categorical	Nature of work
3		Hafiz E Quran (the person remembers the holy book Quran)	Categorical	Yes/No
4		Domicile (it shows the residence area of the person)	Categorical	Area Name
5		Quota (admission based on open merit or local domicile)	Categorical	Open/BWP
6		FSc Board Name (name of intermediate Board)	Categorical	Board Name
7		Entry Test Name (Admission test mandatory for admission)	Categorical	NAT/MCAT
8		Accommodation (whether student living in a hostel?)	Categorical	Yes /No
9		Year of Birth (Year in which the applicant born)	Numeric	Year
10		FSc Passing Year (Intermediate passing year, 12 years of education)	Numeric	Year
11	Academic	FSc Percentage (Percentage marks in Intermediate, 12 years of education)	Numeric	Percentage
12		Entry Test Percentage	Numeric	NAT or MCAT Percentage
13		FSc Urdu Percentage (Percentage marks in Urdu subject in intermediate)	Numeric	Percentage
14		FSc English Percentage (Percentage marks in English subject in intermediate)	Numeric	Percentage
15		FSc Islamic Education Percentage (Percentage marks in Islamic Education subject in intermediate)	Numeric	Percentage
16		FSc Pak Studies Percentage (Percentage marks in Pak Studies subject in intermediate)	Numeric	Percentage
17		FSc Physics Percentage (Percentage marks in Physics subject in intermediate)	Numeric	Percentage
18		FSc Chemistry Percentage (Percentage marks in Chemistry subject in intermediate)	Numeric	Percentage
19		FSc Biology Percentage (Percentage marks in Biology subject in intermediate)	Numeric	Percentage
20		Matric Urdu Percentage (Percentage marks in Urdu subject in matric)	Numeric	Percentage
21		Matric English Percentage (Percentage marks in English subject in matric)	Numeric	Percentage
22		Matric Islamic Education Percentage (Percentage marks in Islamic Education subject in matric)	Numeric	Percentage
23		Matric Pak Studies Percentage (Percentage marks in Pak Studies subject in matric)	Numeric	Percentage
24		Matric Mathematics Percentage (Percentage marks in Mathematics subject in matric)	Numeric	Percentage
25		Matric Physics Percentage (Percentage marks in Physics subject in matric)	Numeric	Percentage
26		Matric Chemistry Percentage (Percentage marks in Chemistry subject in matric)	Numeric	Percentage
27		Matric Biology Percentage (Percentage marks in Biology subject in matric)	Numeric	Percentage
28		Matric Percentage (Percentage marks in Matric, 10 years of education)	Numeric	Percentage
29		SGPA (First Semester SGPA percentage)	Numeric	Percentage
30		SGPA (final semester SGPA, 0/1 for binary classification models where 0 indicate SGPA < 3 and 1 indicate ≥ = 3.00)	Categorical	0/1(dependant variable)

### Data pre-processing

Python’s SciKit learn and Pandas libraries were used for pre-processing. Some machine learning algorithms don’t work on categorical features, hence categorical features were converted to numeric form using one-hot-encoding where binary valued dummy variables were introduced for each category. Further, due to difference in range values of various numeric/quantitative features some features can influence more while training a machine learning model. To avoid such type of features’ bias, quantitative the features were transformed into same scale where each feature had zero mean and unit variation. The data labelling was performed following^25^ where a student who got at least 3.0 SGPA in the final semester was awarded high performing label as 1, and the rest of the students were awarded as low performing label, 0. The dataset was imbalanced: 150 students belonged to the high-performance category 1 (majority class), and only 16 belonged to the low-performance category 0 (minority class), that can lead to non-generalized machine learning model (aka over fitted model) which perform well on seen/train in data but perform poor on unseen data. The synthetic minority oversampling technique (SMOTE) was used to overcome the imbalanced nature of dataset. Based on a random sampling algorithm, it generated new instances for minority classes using the synthetic sampling technique to create a more balanced distribution. For the minority class, the SMOTE technique selects the examples that are near in features space by drawing a line between examples and drawing a new sample at a point along that line^26^. After SMOTE, the number of data instances raised from 166 up to 300 where each class had 150 samples.

### Predictive modeling and performance evaluation

A supervised machine learning algorithm learns association between records/rows described through independent variables aka features (demographic features, HSC subjects’ marks, HSSC subjects’ marks) and target variable (final semester SGPA, high or low) values as labels (see Table 1). Due to categorical nature of target variable the problem was related to binary class classification.

Five (05) supervised classification algorithms popular in the literature were utilized to build prediction models. A decision tree is a supervised machine learning classification algorithm based on the divide and conquers concept. It is like a structured flowchart, where the data/features are divided into root node and child nodes as per feature selection criteria. The process starts from the root node as a highly valuable feature for prediction the target variable, then a child node is created for each subset. This process is repeated until the leaf node is found^27^. But it is prone to overfitting that can be minimized using early stopping in training phase or post pruning after training the model. An over-fitted model memorizes the training samples very well but produces poor generalization on unseen data. To reduce the overfitting, the Random Forest algorithm combines the results of various decision trees by majority voting. In a Random Forest, each decision tree is generated by considering a random sample of attributes. Every decision tree produces a classification for each object, called “vote” for that class. The random forest assigns to each object the class having a higher number of votes^28^. The Support Vector Machine (SVM) algorithm is based on the structural risk minimization principle. It is a statistical approach used to divide the dataset into two classes according to the hyperplane which has the maximum distance to the nearest support vector (data point) of any class^29^. It is effective due to its performance^30^. The classification algorithm, K-Nearest Neighbours (KNN) is popular due to its simplicity and effectiveness. In KNN, data is classified according to k-neighboring data points. Classification is based on the majority of voting among the neighboring data points. Best K plays an important role in classification^31^. Logistic Regression (LR) is a statistical model based on the logistic function to model binary dependent variables. It predicts probabilities of the dependent variable for the combination of independent variables and is used to determine the combination of best independent predicted variables^32^.

To increase a model’s generalizability (or to avoid over fitting), a three-step approach was performed. First, we implemented SMOTE (discussed earlier) to overcome imbalanced dataset problems. Second, a recursive feature elimination (RFE) method was used for optimal features selection. RFE is a most commonly used wrapper approach^33^, which selects features based on machine learning model performance. Third, hyper parameter tuning was performed using grid search. SciKit learn library provides the GridsearchCV function for parameter tuning to determine the optimal values for a given prediction model. The function evaluates the model for each combination of parameters specified in a grid. Four parameters of the GridsearchCV were used in this study: estimator (aka classifier), parameter grid- list of values of estimator parameters, cross-validation, and scoring to measure the performance.

To evaluate supervised classification prediction models, three (03) well-known evaluation metrics were used: precision, recall, and accuracy.

### Rule generation and feature extraction

The decision rules were generated based on a decision tree to get the performance affecting features. By looking at the decision tree predictive model, we extracted rules and identified key features by traversing the parts of paths of the decision tree^34^ that leads to the nodes labeled as high or low-performing students. The extracted rules and key features can be interpreted by faculty and administration for benefits of students and policy making.

## Results and discussion

The experiment related to machine learning were performed using python’s SciKit learn library. The dataset was partitioned into 15 folds cross-validation: 85% training and 15% testing datasets, k-number of times. This sampling method is useful to overcome overfitting specifically when the dataset is in small size. The results are shown and discussed according to the research questions.

RQ1: Can we predict the final semester performance of a Doctor of Veterinary Medicine (DVM) student with high accuracy based on pre-admission features and first-semester performance?

Five supervised machine learning algorithms were used and their performance was evaluated using three metrics: Precision, Recall, and Accuracy (See Table 2). The model based on SVM produced best performance in all the three metrics, followed by Random Forest, and Decision Tree. Note that for the top-3 performance prediction models, Precision and Recall were high and almost had similar results, which shows models were predicting performance of both types of students (low or high performing) with equal confidence. That is predictive models are quite capable to predict performance of low and performing students.

**Table 2:** Students’ performance prediction models based on 15-folds cross validation results.

Metric	Classification algorithm				
	Decision tree (%)	Random forest	Support vector machine (%)	K-nearest neighbours (%)	Logistic regression (%)
Precision	80	87	**93**	81	72
Recall	80	86	**92**	70	72
Accuracy	80	86	**92**	67	72

Top result values are in bold.

RQ2: What are the features that affect the final semester performance of the DVM student?

**Figure 2:**
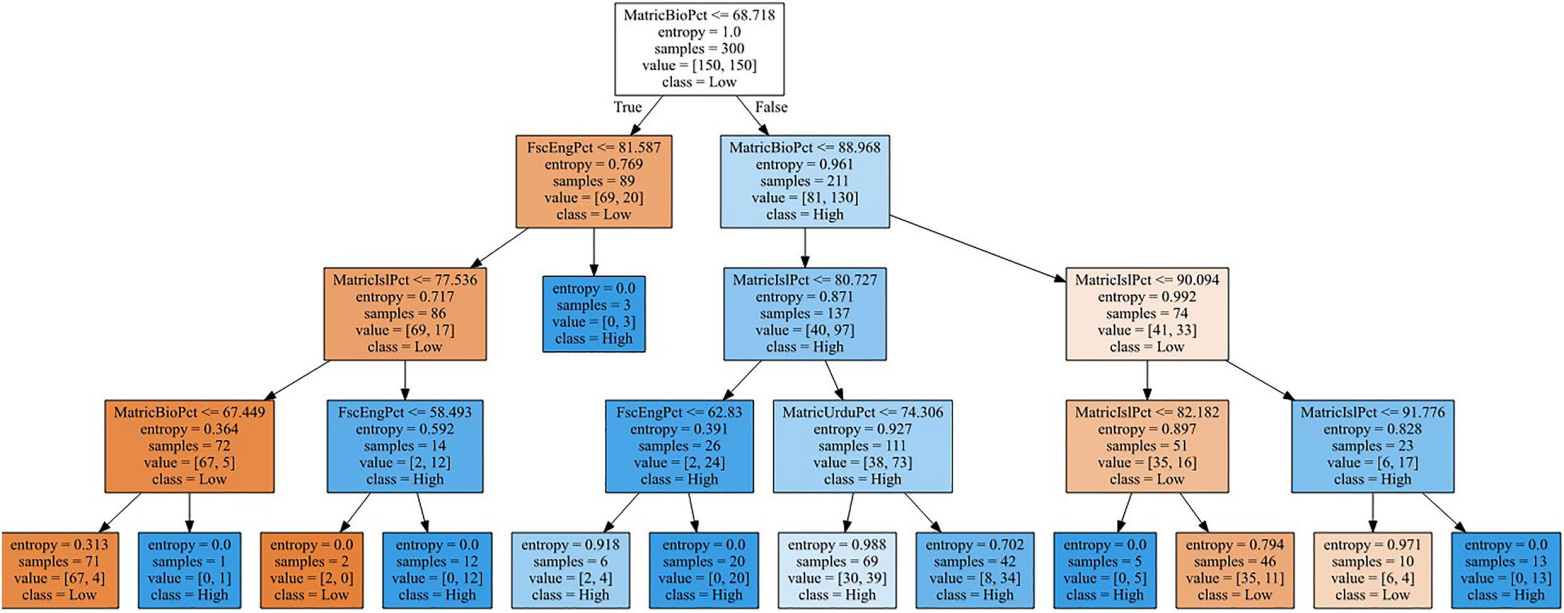
Hierarchical model of a decision tree where the label, high shows a student had at least 3.00 SGPA in the final semester results.

Five classification algorithms were used to predict students’ performance. These classifiers, except decision tree, are not easily interpretable by humans. In this study, performance (80%) of decision tree is low as compared to Random Forest and Support Vector Machine but this hierarchical model (Fig. 2) is interpretable where each node is feature. The root node is top quality feature. The decision rules (See Table 3) were generated by traversing the paths of the decision tree of Fig. 2, top to bottom Using decision tree and its associated rules by following the study^34^, we also extracted performance affecting features^34^ for high or low performing students:

1. In matriculation, students with marks greater than 69% in Biology or students with marks greater than 91% in Islamiat, were likely to fall in high performers in the final semester.
2. In intermediate, students with marks less than or equal to 58% in English, were likely to have fall in low performers in the last semester.

**Table 3:** Decision rules derived from a decision tree, where values are % marks in different subjects.

Sr. No	If Conditions	THEN Class
1	MatricBioPct < = 68.72 AND FscEngPct < = 81.59 AND MatricIslPct < = 77.54 AND MatricBioPct < = 67.45	Class 0
2	MatricBioPct < = 68.72 AND FscEngPct < = 81.59 AND MatricIslPct < = 77.54 AND MatricBioPct > 67.45	Class 1
3	MatricBioPct < = 68.72 AND FscEngPct < = 81.59 AND MatricIslPct > 77.54 AND FscEngPct < = 58.49	Class 0
4	MatricBioPct < = 68.72 AND FscEngPct (between 77.54 & 81.59 )	Class 1
5	MatricBioPct < = 68.72 AND FscEngPct > 81.59	Class 1
6	MatricBioPct > 68.72 AND MatricBioPct < = 88.97 AND MatricIslPct < = 80.73 AND FscEngPct < OR > 62.83	Class 1
7	MatricBioPct > 68.72 AND MatricBioPct < = 88.97 AND MatricIslPct > 80.73 AND MatricUrduPct < OR > 74.31	Class 1
8	MatricBioPct > 68.72 AND MatricBioPct > 88.97 AND MatricIslPct < = 90.09	Class 1
9	MatricBioPct > 68.72 AND MatricBioPct > 88.97 AND MatricIslPct > 82.18	Class 0
10	MatricBioPct > 68.72 AND MatricBioPct > 88.97 AND MatricIslPct > 91.78	Class 1

Four subjects (Biology, English, Islamiat, and Urdu) were identified as students’ performance affecting features. The three subjects (Biology, Islamiat, and Urdu) belong to matric and one (English) belong to FSc. We can anticipate a student who is interested in Biology will perform better at DVM level due to same nature of subjects. Moreover, good performance in English is also justified for good performance at DVM due to medium of study at DVM was English which is different from native language Urdu. The impact of Islamiat and Urdu subjects on final semester performance is difficult to interpret. A reason may be due to a science student usually don’t take interest in arts related subject and those who took interest in these subjects as well may be more dedicated or hard working students. Low performing students had marks less than 69% in Biology and less than 58% in English. It can also be seen that demographic features did not play an effective role in student performance prediction. This observation is consistent with the observation of some other studies^18,19^ where demographics also didn’t performed in performance prediction, but in some other studies^35–38^ demographic features have shown significant impact on online learning outcomes and students’ performance. The reason of this variation may be due to change in subject, department, geographic location, and native language or varying nature of features used in different studies. Further note that we used decision tree for their interpretability but its performance in this study was 80% whereas other two predictive models reported 92% accuracy. Moreover decision tree based rules are not showing the impact of first semester performance but the experiments (not reported here) without this feature achieved only 76% accuracy.

Our findings are in line with previous studies^14,15,39^ in the sense that academic courses are strong indicators of student performance. Several studies also have suggested the influence of academic features on early academic performance prediction^3,7,14,25,40^. In this study, performance affecting academic features are different from others, and this may be due to the different nature of study discipline.

## Conclusion and future work

In this study, Data Mining Techniques were used to predict students’ final semester academic performance of the DVM undergraduate program using pre-admission features, and the DVM first semester SGPA. The findings of this study can be used to implement some policies. For instance, faculty can take into account performance affecting features to promote better students and provision of additional teaching support to low performing students at early stage. With the aid of expanded experiments, administration can adjust the admission criteria based on performance affecting features on first year results (a future plan of ours). Particularly note that three subjects of matric (Biology, Urdu, and Islamiat) were affecting final semester SGPA which is a new insight in the sense, admission criteria in this part of the world at undergraduate level only consider intermediate performance for merit (not below this) at the time of admission. Based on literature survey and experimentations, it is anticipated that performance affecting features may vary based on specific subject, program, geographical location, nature of study (online or physical), native language. So there is a need to expand the experiments to identify key features for each subject, study program in different part of the world. Seeing the difficulty in data collection and hence in data analysis, digital transformation of academia is recommended.

## Data Availability

The data used in this study are available in anonymized form upon request.
